# The functional ClpXP protease of *Chlamydia trachomatis* requires distinct *clpP* genes from separate genetic loci

**DOI:** 10.1038/s41598-019-50505-5

**Published:** 2019-10-01

**Authors:** Stefan Pan, Imran T. Malik, Dhana Thomy, Beate Henrichfreise, Peter Sass

**Affiliations:** 1 Department of Microbial Bioactive Compounds, Interfaculty Institute of Microbiology and Infection Medicine, University of Tuebingen, Auf der Morgenstelle 28, 72076 Tuebingen, Germany; 2 Institute for Pharmaceutical Microbiology, University of Bonn, Meckenheimer Allee 168, 53115 Bonn, Germany; 3 German Center for Infection Research (DZIF), partner site Tübingen, Tübingen, Germany

**Keywords:** Antibiotics, Bacteriology, Pathogens

## Abstract

Clp proteases play a central role in bacterial physiology and, for some bacterial species, are even essential for survival. Also due to their conservation among bacteria including important human pathogens, Clp proteases have recently attracted considerable attention as antibiotic targets. Here, we functionally reconstituted and characterized the ClpXP protease of *Chlamydia trachomatis* (ctClpXP), an obligate intracellular pathogen and the causative agent of widespread sexually transmitted diseases in humans. Our *in vitro* data show that ctClpXP is formed by a hetero-tetradecameric proteolytic core, composed of two distinct homologs of ClpP (ctClpP1 and ctClpP2), that associates with the unfoldase ctClpX via ctClpP2 for regulated protein degradation. Antibiotics of the ADEP class interfere with protease functions by both preventing the interaction of ctClpX with ctClpP1P2 and activating the otherwise dormant proteolytic core for unregulated proteolysis. Thus, our results reveal molecular insight into ctClpXP function, validating this protease as an antibacterial target.

## Introduction

Bacterial Clp proteases constitute compartmentalized macromolecular machines. On the molecular level, Clp proteases form large complexes that can be separated into two major components: a proteolytic core formed by a barrel-shaped tetradecamer of ClpP subunits^1^ that has to associate with regulatory AAA+ Clp-ATPases (e.g. ClpX and ClpA in *Escherichia coli*, or ClpX and ClpC in *Staphylococcus aureus*) to allow for substrate recognition and proteolytic activity^2^. In most bacteria including *E*. *coli*, *S*. *aureus* and *Bacillus subtilis*, 14 ClpP monomers arrange as two homo-heptameric rings, which stack vis-à-vis to form a cylindrical structure of about 90 Å in both diameter and height. Inside of the compartmentalized ClpP barrel, a spacious degradation chamber of approx. 50 Å width secludes the active sites of the protease located close to the equatorial plane of the ClpP barrel, which comprise 14 catalytic triads with the canonical residues typical for serine proteases (Ser, His, Asp). The compartmentalized structure of the ClpP tetradecamer effectively shields the active sites from potential protein substrates in the cytoplasmic environment, which can only be accessed by small peptides through narrow entry pores at the apical and distal surfaces of the ClpP barrel. ClpP itself is almost free of substrate specificity and is unable to degrade proteins on its own under natural conditions due to restricted substrate access to the inner proteolytic chamber of the ClpP barrel. Only small peptides that readily diffuse through the entrance pores are degraded^3^. As such, the ClpP tetradecamer by itself should be considered as a peptidase but constitutes the dormant core of the larger proteolytic Clp complex.

For proteolytic activity, the ClpP barrel has to associate with designated Clp-ATPases, hexameric unfoldases, which bind via distinct loops to the buried hydrophobic pockets at the apical sides of the ClpP barrel. The Clp-ATPases select natural Clp substrates, unfold them in an ATP-dependent manner, and thread the unfolded polypeptide chains through the entry pores into the degradation chamber of the ClpP tetradecamer for proteolytic digestion^1,2^. Regarding substrate selection by the corresponding Clp-ATPase, natural Clp substrates are commonly flagged by specific N- or C-terminal degradation tags (so-called degrons)^4–6^. For instance, the 11-amino acid ssrA-tag is attached to the C-terminus of nascent polypeptides in *E*. *coli* when ribosomes stall during translation thereby targeting the erroneous protein for degradation by the ClpXP protease^7^.

On the cellular level, Clp proteases are important for vital cellular functions as they are involved in protein quality control, turnover and homeostasis, i.e. situations in which misfolded or mistranslated proteins accumulate in the cytoplasm due to e.g. heat stress or antibiotic-induced interference of the ribosomal machinery. Moreover, the Clp protease also directs developmental processes like virulence, cell differentiation and genetic competence by temporally and spatially precise proteolysis of regulatory proteins^8–10^. Unsurprisingly so, the Clp protease is conserved across most bacterial species including human pathogens. Some bacteria, such as *Mycobacterium tuberculosis*, *Corynebacterium glutamicum* and *Streptomyces lividans*, even necessitate functional Clp proteases for cell viability^11–14^. Due to their important role in bacterial physiology, and considering the worldwide spread of antibiotic resistance which represents an increasing threat to human and animal health, the bacterial Clp protease emerged as a novel and promising target for antibiotic attack and virulence inhibition^4,15–17^. We here report on both the functioning of the Clp protease of *Chlamydia trachomatis*, an obligate intracellular pathogen and the causative agent of widespread sexually transmitted diseases and trachoma-associated infectious blindness in humans^18,19^, and the deregulation of the chlamydial ClpXP machinery by antibiotic action. Like other pathogenic bacteria, including listeria, clostridia, or pseudomonads^11,20–23^, the genome of *C*. *trachomatis* encodes two *clpP* homologs (*clpP1* and *clpP2*)^24^, that are organized in distinct operons. Also, *C*. *trachomatis* encodes the Clp-ATPases ClpX, ClpC, and the protein-disaggregating chaperone ClpB, the latter, however, is not commonly assumed to interact with ClpP^25^. Our data show that both chlamydial ClpP homologs, ctClpP1 and ctClpP2, are required to form a functional, proteolytic complex when associated with ctClpX, and that antibiotic acyldepsipeptides are capable of deregulating the assembled ctClpXP protease in a dualistic mode of action.

## Results

### Chlamydial ClpP1 and ClpP2 share conserved sequence features despite overall dissimilarity

**Figure 1:**
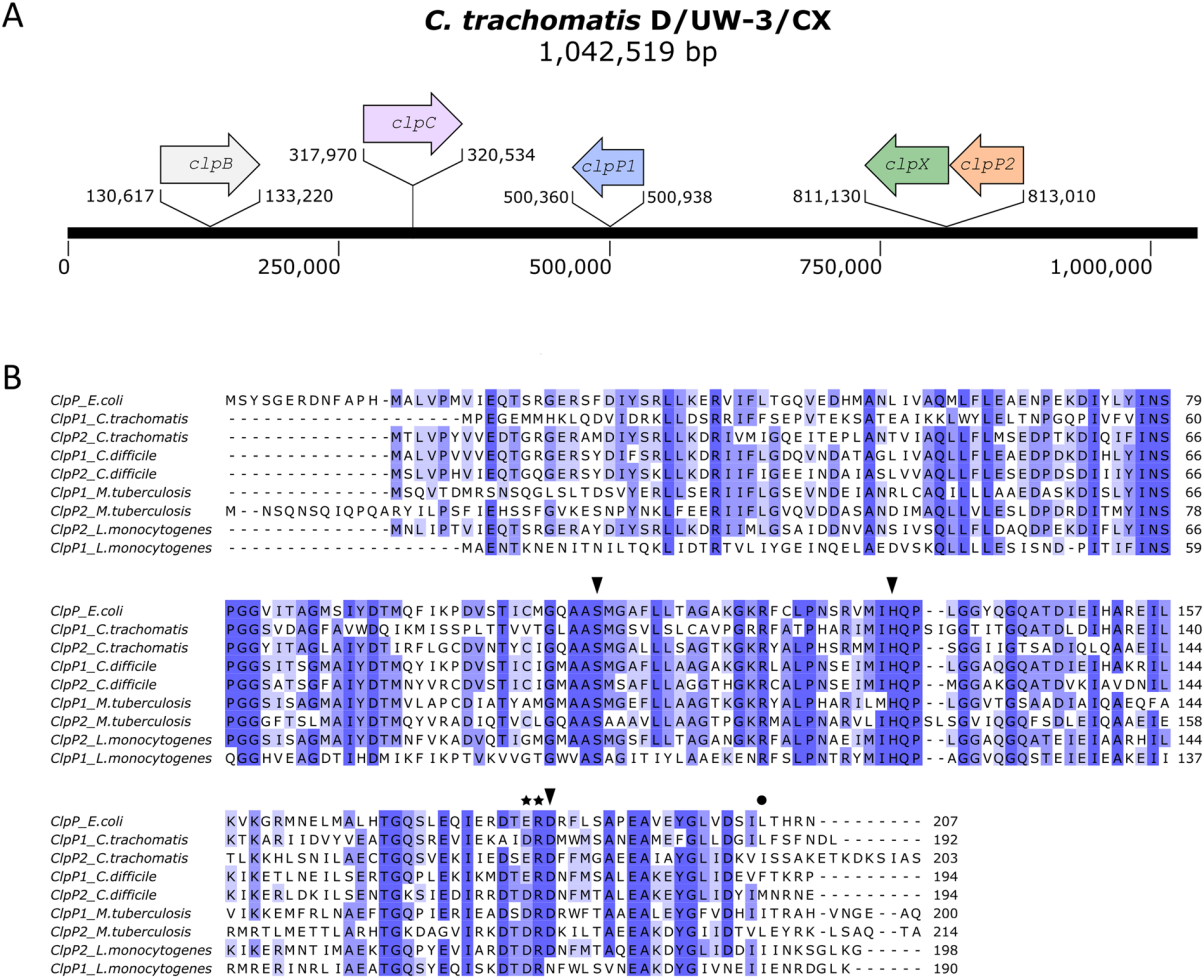
Genomic organization and sequence conservation of chlamydial ctClpP1 and ctClpP2. (**A**) Genetic organization of *clp* genes in the genome of *C*. *trachomatis* D/UW-3/Cx (GenBank NC_000117.1). (**B**) The multi-sequence alignment of ClpP proteins of *Escherichia coli* K-12, *Chlamydia trachomatis* D/UW-3/Cx, *Clostridium difficile* str. 630, *Mycobacterium tuberculosis* H37Rv, and *Listeria monocytogenes* EGD-e was computed using Jalview software^68^. The level of conservation is indicated by a blue scale from dark blue (≥85% similarity) to marine blue (≥65% similarity) to light blue (≥40% similarity). The conserved Ser-His-Asp catalytic triad and the Asp/Arg sensor are marked by black triangles and stars, respectively. The position of hydrophobic pocket mutations constructed in this study (ctClpP1_L186T_ and ctClpP2_I190T_) is indicated by a filled circle.

Previous genome sequencing revealed two putative *clpP* genes in *C*. *trachomatis* (*ctclpP1*, *ctclpP2*)^24^, which are encoded by distinct operons located at different positions of the chlamydial genome (Fig. 1A). For an initial evaluation of putative protease functions, we compared the deduced amino acid sequences of ctClpP1 and ctClpP2 to the thoroughly studied ClpP model protein of *E*. *coli* (ecClpP; UniProtKB P0A6G7, https://www.uniprot.org/) in addition to those of other bacterial species that had previously been described to encode two ClpP homologs (*Clostridium difficile* (cdClpP), *Mycobacterium tuberculosis* (mtClpP) and *Listeria monocytogenes* (lmClpP)) (Fig. 1B). According to the multiple sequence alignment (MSA), central ClpP sequence features such as the Ser-His-Asp catalytic triad^26–28^ as well as the putative oligomeric state Asp/Arg sensor are conserved in ctClpP1 and ctClpP2. Notably, however, while the N-terminal region including the chaperone binding motif^29^ is highly conserved in ctClpP2, ctClpP1 possesses a truncated N-terminal loop region without discernible sequence conservation.

**Figure 2:**
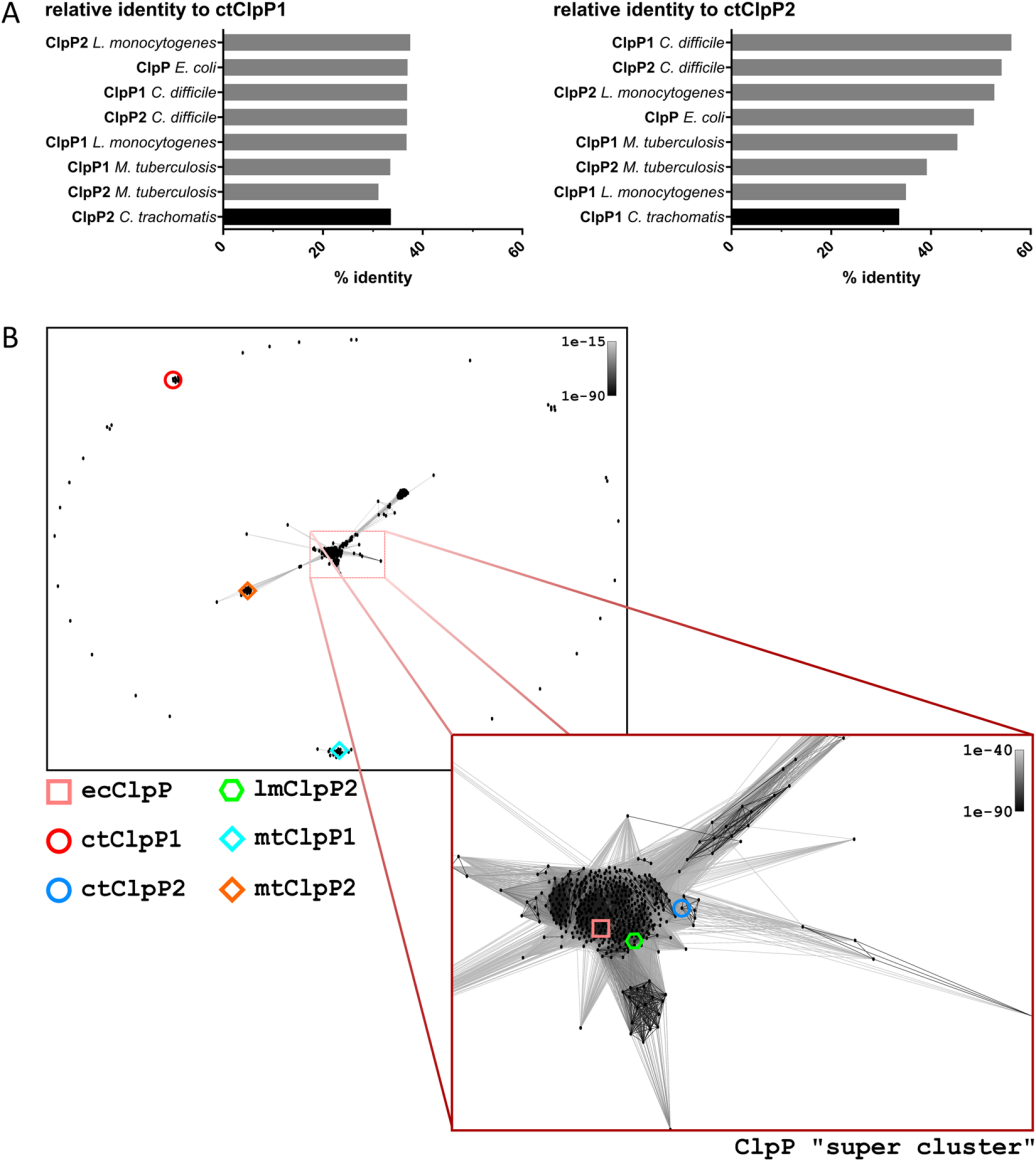
Homology relationships of chlamydial ctClpP1 and ctClpP2. (**A**) Relative identity of ClpP proteins in relation to ctClpP1 (left panel) or ctClpP2 (right panel). (**B**) Homology relationship plots based on CLANS (CLuster ANalysis of Sequences^30^). The P-value threshold was set to 1e-81. ctClpP1 is located in an isolated subgroup containing only ClpP1 of closely related Chlamydia species. Most ClpP protein species including ctClpP2, ecClpP and lmClpP2 reside inside a “super cluster” containing over 70% (437/597) of total input ClpP sequences. Both ClpP homologs of *M*. *tuberculosis* (mtClpP1, mtClpP2) are located at distinct subgroups outside of the “super cluster”.

Regarding homology relationships, the range of overall relative sequence identity of the individual ClpP homologs from these species relative to ctClpP1 was rather narrow (31–37.5%). In contrast, ctClpP2 showed a notably higher variance in sequence identity (33.7–56.2%) (Fig. 2A). In particular, ClpP1 and ClpP2 of *C*. *difficile* or *M*. *tuberculosis* cluster together, suggesting a paralogous relationship. However, ctClpP1 and ctClpP2 showed only low sequence identity to each other, similar to the ClpP homologs of *L. monocytogenes*. In fact, the most dissimilar ClpP homolog in the alignment compared to ctClpP2 is ctClpP1 (33.7%). A homology relationship plot based on CLANS (CLuster ANalysis of Sequences^30^) was calculated to visualize the relationship of 597 unique ClpP species extracted from the UniProtKB/Swiss-Prot database (Fig. 2B). Groups of closest pairs of ClpP homologs were determined by calculating pairwise attraction values based on corresponding High-scoring Segment Pair (HSP) P-values (P < 1E-63). Despite the conserved sequence features that are principally necessary for proteolytic activity, the plot shows that both ClpP isoforms of *C*. *trachomatis* are located at distinct and separate subgroups outside of any densely clustered groups, thereby indicating a non-paralogous relationship of ctClpP1 and ctClpP2.

### ClpP1 and ClpP2 of *C*. *trachomatis* assemble into a hetero-tetradecameric complex that exhibits peptidase activity

**Figure 3:**
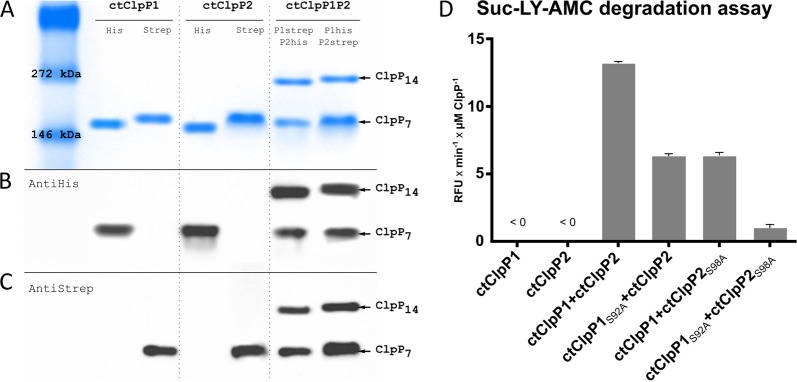
ctClpP1 and ctClpP2 form an active hetero-tetradecameric complex. (**A**) The native PAGE indicates that both ctClpP1 (22.36 kDa) and ctClpP2 (23.25 kDa) form detectable oligomers at a size of roughly 150 kDa, which corresponds to heptameric ClpP complexes (ClpP_7_). When co-incubated (ctClpP1P2), an additional higher molecular weight band of approx. 270 kDa appeared, indicating formation of ctClpP tetradecamers (ClpP_14_). (**B**,**C**) Independent immunodetection using anti-His6-antibodies (**B**) and anti-Strep-antibodies (**C**) reveal both ctClpP1 and ctClpP2 to be present in the newly formed oligomer. (**D**) Peptidase activity assay using Suc-LY-AMC as a substrate for ctClpP1P2. Reaction rates per µM ctClpP in relative fluorescence units per minute (RFU × min^−1^ × µM ClpP^−1^) during the linear phase of substrate degradation of each ctClpP individually and combined are shown. Individually, ctClpP1 and ctClpP2 did not show any detectable peptidase activity. However, when both proteins were combined, ctClpP1P2 efficiently hydrolysed the peptide substrate Suc-LY-AMC. In a mixed situation using catalytic triad deficient derivatives of each ctClpP (ctClpP1_S92A_ and ctClpP2_S98A_) in combination with its respective functional counterpart (ctClpP1 and ctClpP2), the resulting peptidase activity decreased by approx. 60% compared to ctClpP1P2. For ctClpP1_S92A_P2 _S98A_, no significant peptidase activity compared to ctClpP1P2 (<5%) was detected. All experiments were performed as technical triplicates and reproduced using at least three independent biological replicates. The graphs represent one biological replicate with three technical replicates, error bars indicate corresponding standard deviations.

Commonly, the functional ClpP proteolytic core is assembled from two heptameric rings of ClpP proteins, thus requiring a total of 14 ClpP monomers. Regarding bacteria with more than one homolog of ClpP, in principle, tetradecameric ClpP complexes may either be built up by a single ClpP homolog (homo-tetradecamer) or represent mixed complexes of more than one ClpP homolog (hetero-tetradecamer). To determine the composition of chlamydial ClpP complexes, we used native PAGE and immunoblotting techniques to detect C-terminally Strep- and His6-tagged versions of ctClpP1 and ctClpP2 inside assembled complexes. Of note, protein tags did not interfere with ctClpP1 or ctClpP2 oligomerization or activity compared to untagged, wild-type proteins (Figs S1 and S2). For oligomeric state analyses, ctClpPs were used at a final concentration of 7 µM in buffer PZ, incubated at 37 °C for 1 h and were then applied to native PAGE. As shown in Fig. 3A–C, each individual ctClpP homolog formed an oligomer with a respective size of approx. 150 kDa, corresponding to a heptameric state (ClpP_7_). When ctClpP1 was incubated together with ctClpP2 at an equimolar ratio, we detected a second complex roughly double the size compared to individual ctClpPs, indicating the formation of a tetradecameric complex (ClpP_14_). Independent immunodetection using anti-His6- and anti-Strep-antibodies confirmed that both ClpP homologs were present in the larger, tetradecameric complex, leading to the conclusion that ctClpP1 and ctClpP2 interact to form a hetero-tetradecameric complex (ctClpP1P2).

We next examined the peptidase activity of the chlamydial ClpP homologs, individually and combined, using the fluorogenic dipeptide *N*-succinyl-Leu-Tyr-7-amino-4-methylcoumarin (Suc-LY-AMC) as a substrate. The fluorescence signal resulting from peptide cleavage and AMC release was measured. For ctClpP1 and ctClpP2 individually, we did not detect hydrolysis of the peptide substrate. In contrast, when combined, ctClpP1P2 steadily hydrolysed Suc-LY-AMC (Figs 3D, S2 and S3), thus indicating that ctClpP1P2 is the enzymatically functional ctClpP oligomer.

In order to provide further evidence, we investigated whether both ClpP homologs contribute to the enzymatic activity of the assembled complex. To this end, we constructed catalytically deficient derivatives of both ctClpP1 and ctClpP2 by exchanging the catalytic site serine for alanine resulting in ctClpP1_S92A_ and ctClpP2_S98A_, which were then used in Suc-LY-AMC peptidase assays. Indeed, when we substituted either ctClpP1 or ctClpP2 with its mutated counterpart, resulting in the mixed complexes ctClpP1P2_S98A_ or ctClpP1_S92A_P2, the detected peptidase activity decreased by 60% compared to ctClpP1P2 (Figs 3D and S3). As a control, ctClpP1_S92A_P2_S98A_ was almost devoid of peptidase activity compared to ctClpP1P2 (<5%). Of note, mutation of the active site in ctClpP1_S92A_ and ctClpP2_S98A_ did not interfere with oligomerization behaviour of the mutant proteins compared to untagged, wild-type proteins, and thus, loss of activity can be entirely attributed to active site inactivation (Fig. S1). Consequently, both ClpP homologs contributed to peptidase activity of the hetero-tetradecameric complex, thus identifying ctClpP1P2 as the enzymatically functional ctClpP oligomer.

### The ATPase ctClpX confers proteolytic activity to ctClpP1P2

**Figure 4:**
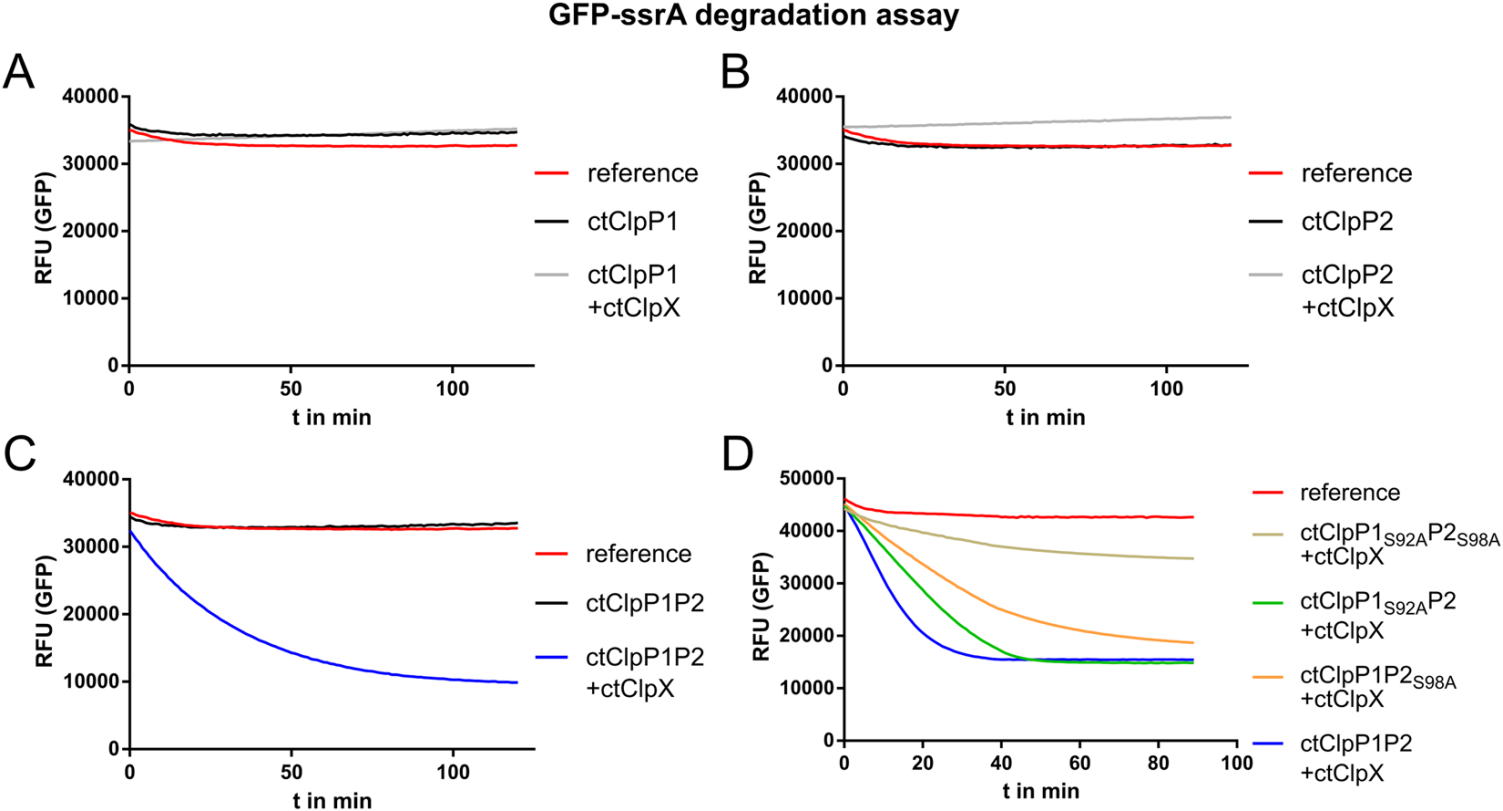
Proteolytic activity of the functional ctClpXP1P2 complex. Time course of GFP-ssrA degradation. (**A**,**B**) In the absence of ClpX, ctClpP1 and ctClpP2 (alone or combined) were devoid of protease activity. Also, addition of ClpX did not trigger protease activity of either ctClpP1 or ctClpP2 alone. (**C**) When ClpX was added to ctClpP1P2, GFP-ssrA was rapidly degraded. (**D**) Proteolytic activity of ctClpXP1P2 was noticeably decreased when ctClpP1 or ctClpP2 were replaced by ctClpP1_S92A_ or ctClpP2_S98A_. Here, the loss of protease activity of ctClpXP1P2_S98A_ was repeatedly stronger than for ctClpXP1_S92A_P2. Reference denotes default GFP fluorescence in buffer solution without enzymes. Results were reproduced using at least three independent biological replicates. Data shown are exemplary for at least three independent experiments.

Since we identified ctClpP1P2 as the enzymatically active complex, we next explored whether the designated, ATP-fuelled Clp-ATPase ClpX of *C*. *trachomatis* (ctClpX) interacts with ctClpP1P2 and confers proteolytic activity to the complex. To this end, we used C-terminally ssrA-tagged GFP (GFP-ssrA) as a model protein substrate. GFP-ssrA is commonly used to measure protease activity of ClpXP complexes, since its proteolysis can be conveniently detected via the fluorescence decrease caused by ClpXP-mediated proteolysis of GFP^7^. In our assays, neither ctClpP1 nor ctClpP2 alone, regardless of the presence of ctClpX, exhibited detectable protease activity. Similarly, also ctClpP1P2 was not capable of degrading GFP-ssrA in the absence of ctClpX. However, when ctClpX was added to ctClpP1P2, we noticed rapid degradation of GFP-ssrA (Fig. 4A–C).

To further verify that both ctClpP homologs are required for protease activity, we substituted ctClpP1 and/or ctClpP2 with the respective catalytic site mutant proteins ctClpP1_S92A_ and ctClpP2_S98A_. In line with our results of the peptidase assay (Figs 3D and S3), substitution of either ClpP1 or ClpP2, or both, lead to a clear decrease of protease activity. In particular, substitution of ctClpP2 with ctClpP2_S98A_ repeatedly showed a stronger effect on proteolytic activity of the complex compared to the substitution of ctClpP1 with ctClpP1_S92A_. As a control, a mixture of ctClpP1_S92A_ and ctClpP2_S98A_ displayed only a slight loss of GFP fluorescence over time in the presence of ctClpX and were regarded inactive (Fig. 4D). Baker and colleagues previously reported a similar behaviour for inactivated ecClpXP^31^, which was attributed to a slow denaturation of GFP-ssrA in the course of ClpX-mediated unfolding and translocation into ClpP with subsequent trapping within the inactive barrel structure. Taken together, our results prove that ctClpP1P2 forms an active proteolytic complex with ClpX.

### ADEP1 inhibits the function of ctClpXP1P2 and triggers independent proteolytic activity of ctClpP1P2

**Figure 5:**
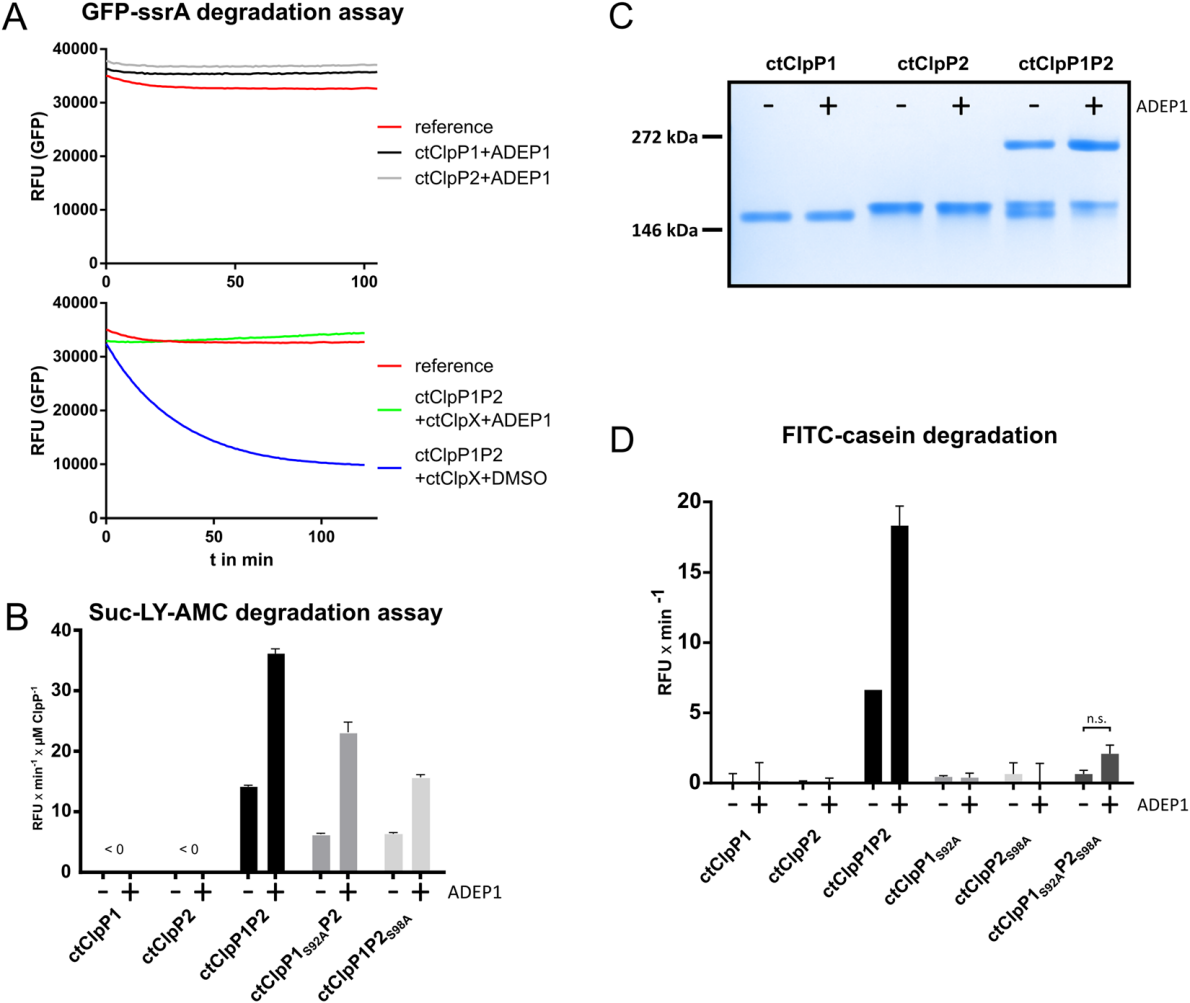
ADEP1 deregulates the chlamydial ClpXP1P2 proteolytic complex. (**A**) ADEP1 inhibits the function of ctClpXP1P2 in GFP-ssrA degradation assays. In the presence of ADEP1, ctClpP1 and ctClpP2 alone remained inactive for GFP-ssrA degradation (upper image). However, ADEP1 abolished GFP-ssrA degradation by ctClpXP1P2 compared to ctClpXP1P2 with DMSO (lower image). Results were reproduced using at least three independent biological replicates. Data shown are exemplary for at least three independent experiments. (**B**) ADEP1 stimulates peptidase activity of ctClpP1P2. Reaction rates per µM ctClpP in relative fluorescence units per minute (RFU × min^−1^ × µM ClpP^−1^) during the linear phase of substrate degradation of each ctClpP individually and combined are shown. Both ctClpP1 and ctClpP2 alone showed no detectable peptidase activity in the absence or presence of ADEP1 as measured via Suc-LY-AMC degradation. However, ADEP1 led to a 2.5-fold increase in peptidase activity of ctClpP1P2. Similarly, ADEP1 stimulated peptidase activity of both ctClpP1_S92A_P2 and ctClpP1P2_S98A_ by 3.6 and 2.5-fold, respectively. (**C**) ADEP1 promotes oligomerization of ctClpP1 and ctClpP2 into tetradecameric ctClpP1P2. No effects were observed for individual ctClpP1 or ctClpP2 proteins in the presence of ADEP1. (**D**) Protease activity assay using FITC-casein as substrate for ctClpP1 and ctClpP2 as well as the respective catalytic triad mutants (ctClpP1_S92A_ and ctClpP2_S98A_). Reaction rates in relative fluorescence units per minute (RFU × min^−1^) during the linear phase of substrate degradation of each ctClpP individually and combined are shown. In accordance to (**B**), ADEP1 failed to activate individual ctClpP proteins to degrade FITC-casein, while protease activity of the mixed ctClpP1P2 complex was stimulated approx. 3-fold in the presence of ADEP1. Analogous to our peptidase activity assay (Fig. 3D), we detected residual protease activity for ctClpP1_S92A_P2_S98A_ (9.6%) compared to ctClpP1P2. Two-sided analysis of variance (two-way ANOVA) determined the apparent ADEP1-stimulated protease activity of ctClpP1_S92A_P2_S98A_ to be non-significant (n.s.). All experiments were performed as technical triplicates and reproduced using at least three independent biological replicates. Graphs represent one biological replicate with three technical replicates; error bars indicate corresponding standard deviations.

Antibiotics of the ADEP class are known to specifically deregulate ClpP, the proteolytic core of the Clp protease complex in bacteria. ADEP binding to ClpP leads to both inhibition of regulated proteolysis by the natural Clp complex (ClpP with associated Clp-ATPase) as well as activation of the dormant core ClpP for the proteolysis of non-native protein substrates^32–39^. In our study, we used the natural compound ADEP1^40^ as a tool to further investigate the function of the ctClpP1P2 complex and to evaluate the potential of chlamydial ClpP as an antibacterial target. First, we addressed the interaction of ctClpP1P2 with ctClpX, since ADEP binding to ClpP has been shown to interfere with ClpX binding in other bacteria^41^. By using the GFP-ssrA assay system, we noticed that ADEP1 did not have an effect on ctClpP1 or ctClpP2 alone (Fig. 5A, upper image), which remained inactive for GFP-ssrA degradation. This was according to our expectations, as GFP-ssrA has not been reported to be targeted by ADEP-activated ClpP for degradation but requires active unfolding by a Clp-ATPase. However, when ADEP1 was added to ctClpXP1P2, the degradation of GFP-ssrA by ctClpXP1P2 was completely inhibited in contrast to the control reaction using DMSO (Fig. 5A, lower image), showing that ADEP1 prevents the normal function of the ctClpXP1P2 complex.

We further investigated the potential for ctClpP1P2 activation by ADEP1 in the absence of ctClpX. In peptidase assays using Suc-LY-AMC, no activation of ctClpP1 or ctClpP2 alone was observed in the presence of ADEP1 (Figs 5B and S4). This outcome is further supported by our observation that even addition of ADEP1 did not facilitate homo-tetradecamer formation of ctClpP1 or ctClpP2 (Fig. 5C), thereby corroborating our results that ctClpP1 and ctClpP2 exist as homo-heptamers and thus lack peptidolytic or proteolytic activity (Fig. 3A). However, ADEP1 noticeably supported hetero-tetradecamer formation of ctClpP1P2 (Fig. 5C) and increased the peptidase activity of ctClpP1P2 by more than 2.5-fold (Fig. 5B). Furthermore, when using a mixed complex of catalytic site mutants, we could show that the overstimulating effect of ADEP1 was repeatedly more pronounced in ctClpP1_S92A_P2 (approx. 3.6-fold) compared to ctClpP1P2_S98A_ (approx. 2.5-fold) (Fig. 5B). Regarding protease activity, we also tested whether ADEP1 activates ctClpP1P2 for degradation of the fluorogenic unfolded model protein substrate fluorescein isothiocyanate-casein (FITC-casein). ADEP1 did not activate either ctClpP1 or ctClpP2 alone for the degradation of the protein substrate (Figs 5D and S5), corroborating our results in Fig. 3D. In contrast, we detected noticeable degradation of FITC-casein by ctClpP1P2 which was further enhanced by ADEP1 (Figs 5D and S5). The unexpected residual activity of ctClpP1P2 to degrade FITC-casein is uncommon for ClpP. As described above, ctClpP1 is characterized by a truncated N-terminal loop region compared to other ClpP homologs (Fig. 1B), and therefore entry of the unfolded protein substrate into the degradation chamber of ctClpP1P2 may be facilitated via a more opened entrance pore of ctClpP1. In this context, N-terminally truncated ecClpP was capable of degrading casein by a mechanism independent of Clp-ATPases as reported earlier^42^. Activation of ctClpP1P2 by ADEP1 was further verified by using unlabelled β-casein as a substrate (Fig. S6). These results confirm the existence of a hetero-tetradecameric complex and show that ADEP1 interferes with the chlamydial Clp protease, leading to both inhibition of ctClpXP1P2 functions as well as activation of ctClpP1P2 for unregulated proteolysis.

### ctClpX and ADEP1 interact with ctClpP2 rather than ctClpP1

Our results showed that the chlamydial Clp-ATPase ctClpX as well as the antibiotic ADEP1 interact with the hetero-tetradecameric complex ctClpP1P2. In principle, ctClpX or ADEP1 may either bind to ctClpP1, ctClpP2, or both in the assembled complex. To further explore the nature of the observed interaction of ctClpX or ADEP1 with ctClpP1P2, we constructed mutant proteins of both ctClpP1 and ctClpP2 by altering their hydrophobic pocket region that is crucial for the binding of ADEPs or the IGF/L loops of ClpX in other bacteria^33,35,41,43,44^.

**Figure 6:**
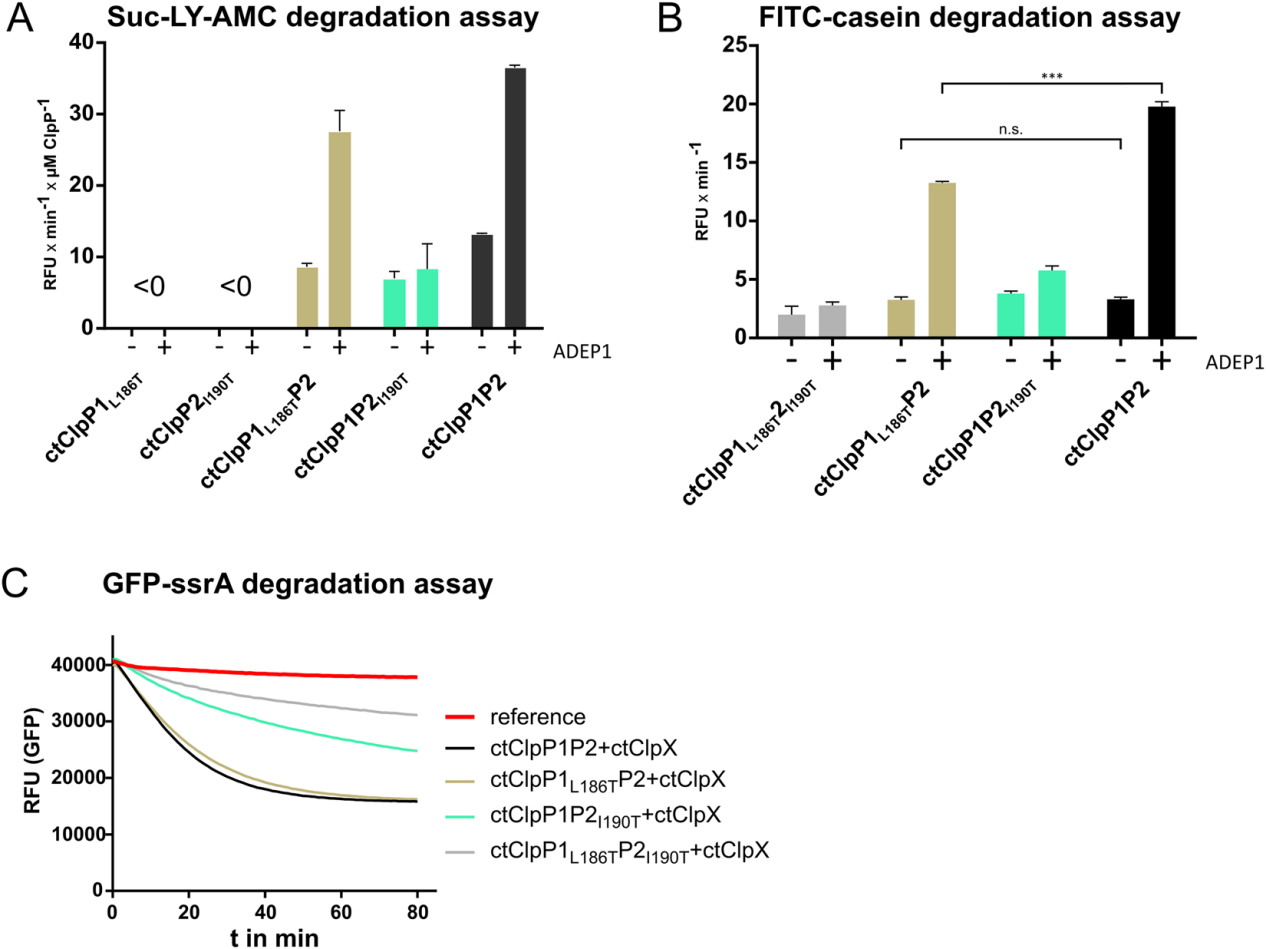
Mutations in the hydrophobic pocket of ctClpP2 prevent activation of the mixed complex by ADEP1 and ctClpX. (**A**) Peptidase activity assay using Suc-LY-AMC as a substrate for wild-type (ctClpP1 and ctClpP2) and hydrophobic pocket mutant proteins (ctClpP1_L186T_ and ctClpP2_I190T_). Reaction rates per µM ctClpP in relative fluorescence units per minute (RFU × min^−1^ × µM ClpP^−1^) during the linear phase of substrate degradation of each ctClpP individually and combined are shown. Here, ctClpP1_L186T_ or ctClpP2_I190T_ alone did not show any detectable peptidase activity. However, in a mixed situation using ctClpP1_L186T_P2, ADEP1 clearly stimulated peptidase activity almost to wild-type level (ctClpP1P2), in contrast to the mixed complex of ctClpP1P2_I190T_. All experiments were performed as technical triplicates and reproduced using at least three independent biological replicates, error bars indicate corresponding standard deviations. (**B**) FITC-casein degradation assay using wild-type ctClpP proteins (ctClpP1 and ctClpP2) and corresponding hydrophobic pocket mutant proteins (ctClpP1_L186T_ and ctClpP2_I190T_). Reaction rates in relative fluorescence units per minute (RFU × min^−1^) during the linear phase of substrate degradation of each ctClpP individually and combined are shown. Here, ADEP1 clearly stimulated the protease activity of the mixed complex ctClpP1_L186T_P2 in a similar fashion as wild-type ctClpP1P2. However, only weak activation was observed for ctClpP1P2_I190T_, indicating preferred interaction of ADEP1 with ctClpP2. All experiments were performed as technical triplicates and reproduced using at least three independent biological replicates. The graphs represent one biological replicate with three technical replicates, error bars indicate corresponding standard deviations. The statistics of ctClpP1P2 compared to ctClpP1_L186T_P2 were performed via two-way ANOVA determination (***p > 0.001; n.s., not significant). (**C**) Time course of ctClpX-dependent degradation of GFP-ssrA using combinations of ctClpP wild-type and hydrophobic pocket mutant proteins. Degradation of GFP-ssrA by ctClpX in combination with either ctClpP1_L186T_P2_I190T_ or ctClpP1P2_I190T_ was noticeably decreased. However, a combination of ctClpX with ctClpP1_L186T_P2 was similarly active to wild-type ctClpP1P2, indicating interaction of ctClpX with ctClpP2. Data shown are exemplary for at least three independent experiments using at least three independent biological replicates.

We first examined these mutant proteins, ctClpP1_L186T_ and ClpP2_I190T_, regarding their potential activation by ADEP1. In Suc-LY-AMC peptide degradation assays (Figs 6A and S7), addition of ADEP1 did not confer peptidase activity to either ctClpP1_L186T_ or ClpP2_I190T_ alone, similar to the non-mutated proteins (Fig. 5B**)**. Intriguingly, ADEP1 also did not affect peptide degradation by ctClpP1P2_I190T_. However, peptidase activity of ctClpP1_L186T_P2 was clearly stimulated to an almost similar level as wild-type ctClpP1P2 in the presence of ADEP1. Although we cannot exclude a weak binding of ADEP1 to ctClpP1, our result indicates that ADEP1 preferably binds to ctClpP2 rather than to ctClpP1. Corroborating this result on the level of protease activity, ADEP1 clearly stimulated the degradation of FITC-casein by ctClpP1_L186T_P2, but only a weak effect was observed with ctClpP1P2_I190T_ (Figs 6B and S8).

We next studied the impact of the hydrophobic pocket mutations on the interaction with ctClpX using GFP-ssrA degradation assays (Fig. 6C). According to our results with ADEP1, GFP-ssrA degradation was clearly impaired for ctClpX in combination with ctClpP1P2_I190T_, so was ctClpP1_L186T_P2_I190T_. In contrast, ctClpX stimulated GFP-ssrA degradation by ctClpP1_L186T_P2 similar to wild-type ctClpP1P2, thus implying a preferential interaction of ctClpX with ctClpP2 of the mixed protease complex.

## Discussion

*C*. *trachomatis* is an obligate intracellular pathogen, which causes sexually transmitted urogenital infections as well as ocular infections that can lead to trachoma, a major cause of preventable blindness in developing countries^18,19,45,46^. The intracellular lifestyle of chlamydiae and a lack of tools for molecular genetic manipulation greatly hampered research on this pathogen during the last decades; however, development of new means to treat and prevent diseases caused by *Chlamydia* requires a fundamental understanding of the biology of this pathogen. The first published genome of *C*. *trachomatis* (strain D/UW-3/CX) allowed initial insights into the biology of *Chlamydia* as it revealed a rather small genome size of 1,042,519 base pairs along with a remarkable reduction of biosynthetic capacities^24,45,47^. This is assumed to be the result of reductive evolution, which emerges as a common feature among *Chlamydia* and other obligatory intracellular parasites^48,49^. As such, the total gene repertoire of these organisms tend to undergo either adaptive genome “streamlining” or simply gene elimination^50^. With that in mind, the existence of two homologous *clpP* genes in *Chlamydia* is striking, indicating a possible importance for the chlamydial lifestyle. Indeed, multiple ClpP isoforms in bacteria are comparatively rare and have been shown to be essential for virulence or even cell viability^12,51,52^.

In this study, we characterized the ClpXP protease of *C*. *trachomatis* in terms of its molecular organization and enzymatic capabilities. The genes encoding ctClpP1 and ctClpP2 are located on different operons at distant regions of the genome. Furthermore, we observed an overall low sequence identity of ctClpP1 compared to ctClpP2, despite the presence of central ClpP sequence features in both homologs that are important for proteolytic activity, such as the Ser-His-Asp catalytic triad or the Asp(Glu)/Arg oligomerization sensor domain, a strong predictor for ClpP tetradecamer assembly^27,28,53^. In principle, the genetic organization and the conserved sequence features allow for two putative hypotheses on the composition and function of the chlamydial Clp protease: i) distinct and independent protease functions mediated by two different types of homo-tetradecameric proteolytic cores made of either ctClpP1 or ctClpP2, or alternatively, although not mutually exclusive, ii) joint activity of both ctClpP homologs organized in a hetero-tetradecameric complex (ctClpP1P2).

To address these questions, we heterologously overexpressed the chlamydial Clp proteins ctClpP1, ctClpP2, and the Clp-ATPase ctClpX in *E*. *coli* dAPX-1 (Δ*clpXPA*; Fig. S9) and then characterized the purified proteins using a variety of molecular and enzymatic *in vitro* assays. In our study, ctClpP1 and ctClpP2 individually formed homo-heptameric structures, but no homo-tetradecameric complexes. The latter is generally regarded as a prerequisite for an active Clp protease complex^4,43,44^. Consistent with this, ctClpP1 or ctClpP2 alone did not show independent peptidase or protease activity in our assays and could also not be activated by the natural partner Clp-ATPase ctClpX or by the antibiotic ADEP1. Quite the contrary, we detected tetradecameric complexes only by combining both ctClpP1 and ctClpP2. When mixed, ctClpP1 and ctClpP2 were capable of degrading the peptide substrate Suc-LY-AMC as well as protein substrates such as GFP-ssrA (in combination with ctClpX) or FITC-casein. Using the catalytic triad mutant proteins ctClpP1_S92A_ and ctClpP2_S98A_, we could further show that both ctClpP homologs contributed to the overall activity of the hetero-tetradecameric complex, indicating a functional alignment of the active site residues in both ctClpP homologs. Our findings thus conclusively reveal the existence of a functional hetero-tetradecameric ctClpP1P2 complex in *C*. *trachomatis* that most probably consists of two stacked rings of each a ctClpP1 and a ctClpP2 homo-heptamer.

While our study was in progress, another group very recently reported on the initial characterization of ClpP homologs of *C*. *trachomatis* via a series of life-cell and *in vitro* assays^54^. In that study, however, a different model for the composition and function of ctClpP1 and ctClpP2 complexes was proposed, reporting ctClpP1 to adopt an inactive heptameric conformation and ctClpP2 alone to assemble into proteolytically active homo-tetradecamers *in vitro*. The authors therefore depicted a model that differs from our findings, which we think could be for the following reasons. Aside from thoroughly conducted and conclusive life-cell experiments, which corroborate our finding that ctClpP1 and ctClpP2 both form homo-heptameric structures, the authors used *E*. *coli* BL21 (DE3) to produce recombinant ctClpP proteins for their *in vitro* assays and a potential contamination by endogenous *E*. *coli* Clp proteins during protein purification and in subsequent assays cannot be entirely excluded. Cross-contamination with ecClp proteins in particular may misguide experiments using ClpP proteins with significantly less activity than ecClpP, such as ctClpP1 or ctClpP2. As previously reported for the expression of *C*. *difficile* ClpP proteins using *E*. *coli* BL21(DE3), endogenous ecClpP was detected with a relative abundance of 10–30% of the total purified ClpP proteins^22^. Also in our hands, when we attempted to express ctClpP1 and ctClpP2 in *E*. *coli* BL21 (DE3), contamination with ecClp proteins was observed (data not shown). Since cdClpP1, cdClpP2 as well as ctClpP2, but not ctClpP1, share high sequence identity with ecClpP (Fig. 2A), co-purified ecClp proteins may interfere with oligomerization behaviour and activity in addition to differences in assay set-up, buffer systems and protein purification methods. To exclude co-purification of ecClp proteins, we used the triple-knockout mutant strain *E*. *coli* dAPX-1 as an expression host in which we had deleted the genes encoding ecClpP, ecClpX and ecClpA. Absence of endogenous ecClpP was verified by immunodetection (Fig. S9). We further added ctClpX in our studies, thereby providing a comprehensive and conclusive picture of a fully assembled ClpXP protease complex of *C*. *trachomatis*.

Our data shows that the protease function of ctClpP1P2 in the natural context depends on a native partner Clp-ATPase, here ctClpX, which accords to findings for most bacterial ClpP homologs so far^29,55–57^. These results suggest that the N-terminal loop regions surrounding the axial pore of ctClpP1P2 serve as a hydrophobic plug to close the pore channel similar to ecClpP^36^, thereby preventing influx of folded proteins or complex, large peptide chains. *C*. *trachomatis* encodes three putative Clp-ATPases (ctClpB, ctClpC, and ctClpX), however, only ctClpC and ctClpX possess the conserved (L/I/V)-G-(F/L) tripeptide motif which is required for binding to the hydrophobic pockets of the ClpP tetradecamer^58–61^. In our study, we characterized the role of ctClpX and it emerged that, aside from principally conferring proteolytic activity to ctClpP1P2, ctClpX (as well as ADEP1) preferably interacted with ctClpP2 of the mixed, assembled ctClpP1P2 complex, as indicated by the results obtained from the hydrophobic pocket mutants ctClpP1_L186T_ and ctClpP2_I190T_. This corresponding ADEP1 binding position inside the hydrophobic pocket (Leu189) has been previously inferred from co-crystallization of ADEP1 with ecClpP and bsClpP, respectively^33,35^. Our observed ctClpX binding preference for ctClpP2 is supported by the genetic organization of the *ctclpX* and *ctclpP2* genes as one operon and the truncated nature of the N-terminal region of ctClpP1, potentially hampering the interaction with Clp-ATPases and ADEP1, respectively (Fig. 1A,B). Indeed, the deletion of a similar number (4–6) of amino acids from the N-terminal region of ecClpP abrogated its interaction with ecClpA^42^.

In previous studies, we and others have reported that antibiotics of the ADEP class interfere with the activity of the Clp protease of different organisms^32,33,35,37,41,56,62–65^. For a comprehensive review on the activation of ClpP by ADEP antibiotics, we would like to refer the reader to Malik and Brötz-Oesterhelt^43^. Like Clp-ATPases, ADEPs bind to the same hydrophobic pocket region of the ClpP tetradecamer, thereby abrogating the interaction of ClpP with its partner Clp-ATPase and inhibiting all physiological functions of the Clp protease in natural proteolysis^34,37,41^. At the same time, ADEP activates and confers independent proteolytic activity to the otherwise dormant core ClpP, leading to the unnatural proteolysis of nascent peptides and essential proteins^34,38,66^. In the current study, we used ADEP1 to further characterize the chlamydial Clp system. We observed that ADEP1 led to the activation of both peptidase and protease activity of ctClpP1P2 in the absence of ctClpX, while it had no effect on either ctClpP1 or ctClpP2 alone. In addition, ADEP1 abrogated GFP-ssrA degradation by ctClpXP1P2, indicating that ADEP1 is capable of displacing ctClpX from the ctClpP1P2 complex, eventually inhibiting natural ctClpXP1P2 functions. Like ctClpX, ADEP1 appeared to preferably bind to ctClpP1P2 via ctClpP2. These results corroborate our findings that ctClpP1P2 interacts with ctClpX for protein degradation and further identify the chlamydial Clp protease as a potential target for antibiotic attack by ADEP.

Our data reveal the composition of a functionally active Clp protease complex of *C*. *trachomatis*, which required a hetero-tetradecameric ClpP core of both ctClpP1 and ctClpP2 to interact with ctClpX for regulated protein degradation. A similar organisation of the ClpP protease complex is found in *M*. *tuberculosis* of which two distinct mtClpP proteins (mtClpP1 and mtClpP2) are required to form an active hetero-tetradecameric complex^20^. On the genomic level, however, *mtclpP* genes differ from *Chlamydia* as they are organized in a single operon and are co-transcribed. In contrast, the genes encoding ctClpP1 and ctClpP2 are located on different operons at distant loci of the chromosome, similar to the *clpP1* and *clpP2* genes from *Pseudomonas aeruginosa*, *C*. *difficile* or *L*. *monocytogenes*^21–23^. In addition, ctClpP1 and ctClpP2 share rather low amino acid sequence identity, even when compared to homologous ClpP proteins from a variety of unrelated, multi-ClpP species. Such genetic organization paired with a low amino acid sequence identity might suggest separate, distinct functions for the individual ctClpP proteins. However, our data reveals a joint function of both ctClpP homologs, implying that the genetic organization as well as the sequence identity do not serve as reliable indicators to predict Clp protease complex formation and function.

**Figure 7:**
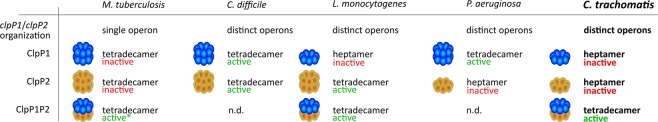
Overview of Clp protease systems from bacterial species that encode two ClpP homologs. The functional Clp protease complex of *C. trachomatis* is unique among the previously identified Clp systems from other bacteria regarding its genetic organization in combination with its assembled structure and activity *in vitro*. In *M*. *tuberculosis*, mtClpP1 and mtClpP2, encoded by a single bicistronic operon, may either form inactive homo-tetradecamers or functionally active hetero-tetradecamers using the non-natural activator peptide Z-Leu-Leu^20^ (regarded as active*). ClpP homologs from either *C*. *difficile*, *L*. *monocytogenes*, or *P*. *aeruginosa*, which are encoded in distinct operons, may form either functionally active homo-tetradecamers as well as hetero-tetradecamers^21–23^. Thus, in each of these situations, an active ClpP complex can result from the same operon or even a single ClpP homolog. In contrast, chlamydial ClpP homologs are encoded by distinct operons at different locations of the chlamydial genome, and they do not form active homo-tetradecamers, but inactive homo-heptamers. Only the combination of the two homologs from different operons results in the assembly of an active hetero-tetradecamer, which may resemble a safeguard mechanism of ClpP activity on the genomic level.

Regarding genetic organization and enzyme function, chlamydial Clp protease appears unique among the characterized Clp systems in bacteria to date, since two distinct ClpP homologs from different and distant genetic loci have to team up to build a functionally active protease (Fig. 7). It may be hypothesized that such a functional connection of distant genetic loci with regulated proteolysis could play a decisive role in the biology of *Chlamydia*. During the complex life cycle of *Chlamydia*, the described mechanism would ensure that the activity of the Clp protease is only engaged in a situation of efficient replication and transcription of both genomic regions, thus representing a safeguard mechanism of ClpP activity on the genomic level. However, when both ctClpP homologs are available in the chlamydial cell, the ctClpP1P2 complex is characterized by a fail-safe mechanism, since the mutation of the catalytic triad in one of both ctClpP homologs only reduced but did not prevent enzyme function. Thus, functionality of ctClpP1P2 is ensured even when such detrimental mutations occur, underlining a putative importance of Clp protease functions for cell viability in *Chlamydia*.

## Methods

### *In silico* analyses of chlamydial Clp sequences

The multiple sequence alignment (MSA) using ClpP sequences retrieved from UniProtKB database (https://www.uniprot.org/) of *Listeria monocytogenes* EGD-e (Q9RQI6, Q8Y7Y1), *Clostridium difficile* str. 630 (Q180F0, Q180J6), *Mycobacterium tuberculosis* H37Rv (P9WPC5, P9WPC3), *Escherichia coli* K-12 (P0A6G7), and *Chlamydia trachomatis* D/UW-3/Cx (P38002, O84712) was generated using MAFFT (v7.299b; Computational Biology Research Consortium Japan^67^). The resulting MSA was visualized via JalView^68^. Relative sequence identities for each ctClpP were calculated using ‘The Sequence Manipulation Suite’^69^.

Homology relationship plots based on CLANS (CLuster ANalysis of Sequences^30^) were computed using 597 unique ClpP amino acid sequences extracted from the UniProtKB/Swiss-Prot database (reviewed sequences) and the BLOSUM62 Scoring matrix. BLAST HSP E-values up to 1e-20 were extracted for calculating pairwise attraction values. For iterative cluster formation, the P-value threshold was set to 1e-81.

### Construction of an *E*. *coli clpPAX* gene deletion mutant for use as protein expression host

To rule out contamination by co-purification of *E*. *coli* ClpP, ClpX, or ClpA proteins during heterologous expression and purification of chlamydial Clp proteins, we constructed a respective *E*. *coli clpPAX* deletion mutant to use as an expression host for protein purification. Here, *E*. *coli* SG1146a (*clpP::camR*) served as the parent strain^7^. Endogenous *clp* gene (*ecclpA*, *ecclpX*) knock outs (KO) were achieved using the lambda Red recombinase system^70^. The kanamycin resistance cassette (*kanR*) of pKD13 served as the target sequence required for recombination selection. For *ecclpX*, a linear KO construct was obtained by PCR using reported primers deposited at Coli Genetic Stock center (CGSC) and pKD13 as template. Resulting mutant *ecclpX::kanR* was then transformed with pCP20 and cured of *kanR* using the FLP recombinase. For *ecclpA*, the KO construct was built using Gibson assembly^71^. Corresponding *ecclpA* flanking regions were obtained by PCR (downstream flanking region: forward primer: 5′ctccagcctacacTCTGATTGTCAGGTAGGTTG′3; reverse primer: 5′taaatacggaaggatctgagatatcGTCGCAGAGTTGGTTACG′3. Upstream flanking region: forward primer: 5′cgcacatttccccgaaaagaagcttCTGTCAGGGTGAAAGAAGGATTTG′3; reverse primer: 5′tcatgtttgacagAGGCACCTCCCCCAATTTTTATG′3). The plasmid backbone was derived from pKD46 (forward primer: 5′gatatcTCAGATCCTTCCG′3; reverse primer: 5′aagcttCTTTTCGGGGAAATG′3). The pKD13 *kanR* fragment was amplified by PCR (forward primer: 5′gggggaggtgcctCTGTCAAACATGAGAATTAATTCCGG′3; reverse primer: 5′cctgacaatcagaGTGTAGGCTGGAGCTGCTTC′3). The resulting *E*. *coli* BL21 (DE3) mutant features Δ*clpX clpP::cam clpA::kan* and is referred to as *E*. *coli* dAPX-1. Loss of ClpP expression was re-confirmed by immunoblotting using anti-ClpP antibodies (Fig. S9).

### Bacterial strains and growth conditions

*E*. *coli* cells were grown in lysogeny broth (LB) at 37 °C under vigorous shaking. When appropriate, the growth medium was supplemented with the antibiotic ampicillin (100 µg/ml) or the inducing compound isopropyl β-D-1-thiogalactopyranoside (IPTG, 1 mM final concentration) for protein expression. All recombinant Clp proteins of *C*. *trachomatis* were expressed using *E*. *coli* dAPX-1 to prevent the co-purification of *E*. *coli* Clp proteins.

### Plasmid construction for the expression of Clp proteins

Plasmids for the expression of *clpP* genes of *C*. *trachomatis* D/UW-3/CX (GenBank: NC_000117; *ctclpP1*, orf CT_431; *ctclpP2*, orf CT_706) were obtained by ligating PCR amplified polynucleotides containing C-terminally Strep-tagged or His6-tagged *ctclpP1* or *ctclpP2* into the linearized pET-11a vector (Merck). A C-terminal Strep-tag sequence was added to *ctclpP1* and *ctclpP2* by cloning each respective gene into pASK-IBA2C using *Bsa*I restriction sites and the following primers: *ctclpP1-*for 5′ATGGTAGGTCTCAGGCCATGCCTGAAGGGGAAATGATG′3, *ctclpP1-*rev 5′ATGGTAGGTCTCAGCGCTCAAGTCGTTAAAAGAGAAGAGAATC′3, *ctclpP2-*for 5′ATGGTAGGTCTCAGGCCATGACGTTAGTACCATACGTTG′3, *ctclpP2-*rev 5′ATGGTAGGTCTCAGCGCTAGACGCAATACTCTTATCTTTTG′3. Subsequently, *ctclpP1 or ctclpP2* inserts containing Strep-tag sequences were amplified by PCR (*ctclpP1strep-*for 5′ATGCATATGCCTGAAGGGGAAATGATG′3, *ctclpP1strep-*rev 5′ATGGGATCCTTATTATTTTTCGAACTGCGG′3, *ctclpP2strep-*for 5′ATGCATATGACGTTAGTACCATACGTTG′3, *ctclpP2strep-*rev 5′ATGGGATCCTTATTATTTTTCGAACTGCGG′3) and cloned into pET-11a using *Nde*I and *Bam*HI. In the same manner, *ctclpX* (GenBank: NC_000117; orf CT_705) was first cloned into pASK-IBA2C (*ctclpX*-for 5′ATGGTAGGTCTCAGGCCATGACAAAAAAAAATCTTGCGG′3, *ctclpX*-rev 5′ATGGTAGGTCTCAGCGCTAGCAATCGCCTCTGGTGATT′3) using *Bsa*I and amplified via PCR (*ctclpXstrep*-for 5′ATGCATATGACAAAAAAAAATCTTGCGG′3, *ctclpXstrep*-rev 5′ATGAAGCTTTTATTATTTTTCGAACTG′3) to obtain the *ctclpXstrep* insert element which was then cloned into pET-22b using *Nde*I and *Hind*III restriction sites. For the expression of ctClpP1-6His, *ctclpP1* was cloned into pET-22b*NcoI (pET-22b vector containing an additional in-frame *Nco*I restriction site upstream of the 6His sequence) using *Nde*I and *Nco*I restriction sites and the following primers: *ctclpP1-6His-*for 5′ATGCATATGCCTGAAGGGGAAATGATG′3, *ctclpP1-6His* rev 5′ATGCCATGGCAAGTCGTTAAAAGAGAAGAGAATC′3. For ctClpP2-6His, *ctclpP2* was cloned into pET-22b using *Nde*I and *Xho*I restriction sites and the following primers: *ctclpP2-6His-*for 5′ATGCATATGACGTTAGTACCATACGTTGTTG′3, *ctclpP2-6His-*rev 5′ATGCTCGAGAGACGCAATACTCTTATCTTTTG′3. Construction of plasmids and sequence identity of *clp* genes were verified by Sanger sequencing (LGC genomics, Germany) followed by pairwise alignments compared to the *clp* genes of *C*. *trachomatis* D/UW-3/CX (NC_000117.1).

### Site-directed mutagenesis

Targeted mutagenesis of the ctClpP catalytic site serine to alanine (ctClpP1_S92A_, ctClpP2_S98A_) was achieved via the “QuickChange II Site-directed mutagenesis” kit (Agilent) according to the manufacturers protocol using the following primers: *ctclpP1*_S92A_-for 5′ggatctgtattgGCAttgtgtgctgttc′3, *ctclpP1*_S92A_-rev 5′gaacagcacacaaTGCcaatacagatcc′3; *ctclpP2*_S98A_-for 5′ggacaagccgctGCAatgggagcgctt′3, *ctclpP2*_S98A_-rev 5′aagcgctcccatTGCagcggcttgtcc′3. In the same manner, mutagenesis of the ctClpP hydrophobic pocket region (ctClpP1_L186T_, ctClpP2_I190T_) was performed using the following primers: *ctclpP1*_L186T_-for 5′tttggactgttagatgggattACCttctcttttaacgacttgagc′3, *ctclpP1*_L186T_-rev 5′gctcaagtcgttaaaagagaaGGTaatcccatctaacagtccaaa′3; *ctclpP2*_I190T_-for 5′tatgggttaattgataaagtgACCtcttctgctaaagagacaaaa′3, *ctclpP2*_I190T_-rev 5′ttttgtctctttagcagaagaGGTcactttatcaattaacccata′3. Mutations were verified by Sanger sequencing (LGC genomics, Germany) followed by pairwise alignments compared to the *clp* genes of *C*. *trachomatis* D/UW-3/CX (NC_000117.1).

### Protein expression and purification

Cultures of *E*. *coli* dAPX-1 harbouring an individual plasmid for the expression of the respective Clp protein were grown until mid-log exponential phase (OD_600_ of 0.6). Then, protein expression was induced by the addition of 1 mM IPTG to the culture, which was further shaken at 18 °C for approx. 16 h until cultures were harvested by centrifugation. All purification steps were executed at 4 °C. Following centrifugation, the cell pellet was resuspended in prechilled buffer A (20 mM Tris/HCl, pH 8). Cell disruption was completed using glass beads (150–212 µm, Sigma) and a Precellys homogenizer based on bead beating technology (Precellys Evolution, Bertin Technologies). Cell debris was pelleted via centrifugation. The remaining supernatant was further filtered using 0.45 µm membrane filters (Sarstedt). Filtered lysates of Strep-tagged proteins were applied to StrepTrap HP 1 ml columns (GE Healthcare). His6-tagged proteins were applied to HisTrap HP 1 ml columns (GE Healthcare). Subsequent protein separation was conducted using the ÄKTA start chromatography system (GE Healthcare) according to the manufacturer’s protocol with following modifications: For His6-tagged proteins, two subsequent washing steps (washing buffer including 20 and 50 mM imidazole) were carried out between sample application and protein elution. Elution fractions containing major target protein amounts were pooled. Protein enrichment and buffer exchange were performed via centrifugal filters (Amicon® Ultracel®-10K, Merck) using buffer GF (20 mM Tris, 5 mM MgCl_2_, 100 mM NaCl, 10% (v/v) Glycerol, pH 7.6). Final purity and concentration of desired proteins were determined via SDS polyacrylamide gel electrophoresis (SDS-PAGE) followed by Bradford assay (Bio-Rad; using BSA as the standard protein), and Nanodrop spectrophotometry (Nanodrop Technologies). C-terminally Strep-tagged ctClp proteins were used in all biochemical assays.

### Native PAGE analyses and immunoblotting

Proteins were used at a final concentration of 7 µM in buffer PZ (25 mM HEPES, 200 mM KCl, 5 mM MgCl_2_, 1 mM DTT, 10% (v/v) glycerol, pH 7.6) and incubated at 37 °C for 1 h. Protein samples were then mixed with native sample buffer (Serva) at a ratio of 1:1, loaded on Novex Tris-glycine ready gels (Thermo), followed by protein separation via electrophoresis at 25 V for approx. 16 h in an ice-cooled chamber. Native PAGE gels were then stained with InstantBlue Coomassie protein stain (Sigma) for visualization of the protein bands.

For immunoblotting, native PAGE gels were incubated for 1 h in transfer buffer (25 mM Tris, 192 mM Glycine, 15% v/v methanol, 0.5% w/v SDS) and subsequently transferred to a PVDF membrane (Merck) via semi-dry protein blotting at 0.8 mA/cm^2^ starting voltage for approx. 80 min according to standard procedures^72^. Immobilized proteins were either probed with mouse-anti-His (IBA, 1:2000) or mouse-anti-Strep (IBA, 1:2000) as primary antibodies followed by rabbit-anti-mouse HRP conjugate (IBA, 1:2000) as secondary antibody. For visualization and detection, ECL Prime Western blotting detection solution (GE) was used as a substrate for the HRP-based secondary antibody, and chemiluminescent signals were detected employing the ChemiDoc documentation system (BioRad).

### Suc-LY-AMC degradation assay

C-terminally Strep-tagged ctClpP proteins were used. In samples containing both ctClpP homologs, equimolar concentrations were used at a 1:1 ratio. Cleavage of the peptide substrate Suc-LY-AMC was carried out in buffer PZ using final concentrations of 3 µM of purified ClpP protein and 100 µM of Suc-LY-AMC (dissolved in DMSO) in 100 µl reaction volumes. Where indicated, a final concentration of 15 µM ADEP1 or the equal volume of DMSO was added accordingly. Reaction samples were pre-incubated for 30 min at 32 °C before the substrate was added to initiate the reaction. Hydrolysis of the peptide was monitored in black, flat-bottom 96-well microplates (Sarstedt) at 32 °C by measuring the release of AMC in a spectrofluorometer (TECAN infinite M200) at excitation wavelength λex = 380 nm and emission wavelength λem = 460 nm. Experiments were performed as technical triplicates; all results were reproduced using at least three independent biological replicates (2^nd^ and 3^rd^ replicates are included in the Supplementary Information section). Data shown represent at least three independent experiments, error bars indicate the corresponding standard deviations.

### FITC-casein degradation assay

Degradation of the fluorogenic protein FITC-casein (fluorescein isothiocyanate-casein; Sigma) was carried out in buffer PZ in 100 µl total volume. Each reaction sample contained final concentrations of 6 μM ctClpP and 20 µM FITC-casein. Where appropriate, a final concentration of 35 µM ADEP1 was added. Equal volumes of DMSO were used for control reactions. Reaction samples were pre-incubated for 30 min at 32 °C before the substrate FITC-casein was added to initiate the reaction. Fluorescence was monitored in black, flat-bottom 96-well microplates (Sarstedt) at 32 °C by measuring the release of FITC in a spectrofluorometer (TECAN infinite M200) at excitation wavelength λex = 490 nm and emission wavelength λem = 525 nm. Experiments were performed as technical triplicates; all results were reproduced using at least three independent biological replicates (2^nd^ and 3^rd^ replicates are included in the Supplementary Information section). Data shown represent at least three independent experiments, error bars indicate the corresponding standard deviations.

### GFP-ssrA degradation assay

Degradation of C-terminally ssrA-tagged eGFP (GFP-ssrA) was carried out in buffer PZ in a total volume of 100 µl. For default fluorescence measurements (reference), a final concentration of 0.36 µM GFP-ssrA in buffer PZ was used. Each reaction sample (except reference) contained final concentrations of 6 μM ctClpX, 7 μM ctClpP, 0.36 μM GFP-ssrA and an artificial ATP regeneration system (4 mM ATP, 8 mM creatine phosphate, and 10 U /ml creatine phosphokinase). Where appropriate, a final concentration of 50 µM ADEP1 or the equal volume of DMSO was then added accordingly. Additionally, in assays using also ctClpP1_S92A_ and ctClpP2_S98A_ (Fig. 4D), the following modification was applied: the final concentration of ctClpP proteins was increased from 7 to 14 µM. All reaction components except for GFP-ssrA were pre-incubated for 30 min at 32 °C before the substrate GFP-ssrA was added. GFP fluorescence was monitored in white, flat bottom 96-well microplates (Greiner) over the course of up to 120 min at excitation wavelength λex = 465 nm and emission at λem = 535 nm using a spectrofluorometer (TECAN infinite M200). Results were reproduced using at least three independent biological replicates. Data shown are exemplary for at least three independent experiments.

Additional datasets generated during and/or analysed during the current study are available from the corresponding author on reasonable request.

## Supplementary information

Supplementary Information
