# Supplementary material for: The functional ClpXP protease of Chlamydia trachomatis requires distinct clpP genes from separate genetic loci
Source: Sci Rep. 2019 Oct 1;9:14129. doi: 10.1038/s41598-019-50505-5 (PMC6773864; doi:10.1038/s41598-019-50505-5)
Supplement: Supplementary file 1 — Supplementary Information [file 41598_2019_50505_MOESM1_ESM.pdf]

SUPPLEMENTARY INFORMATION

The functional ClpXP protease of *Chlamydia trachomatis* requires distinct *clpP* genes from separate genetic loci

Stefan Pan[1], Imran T. Malik[1], Dhana Thomy[1], Beate Henrichfreise [2], Peter Sass[1,3*]

[1]Department of Microbial Bioactive Compounds, Interfaculty Institute of Microbiology and Infection Medicine, University of Tuebingen, Auf der Morgenstelle 28, 72076 Tuebingen, Germany

[2]Institute for Pharmaceutical Microbiology, University of Bonn, Meckenheimer Allee 168, 53115 Bonn, Germany

[3]German Center for Infection Research (DZIF), partner site Tübingen, Tübingen, Germany

[*]Correspondence should be addressed to peter.sass@uni-tuebingen.de

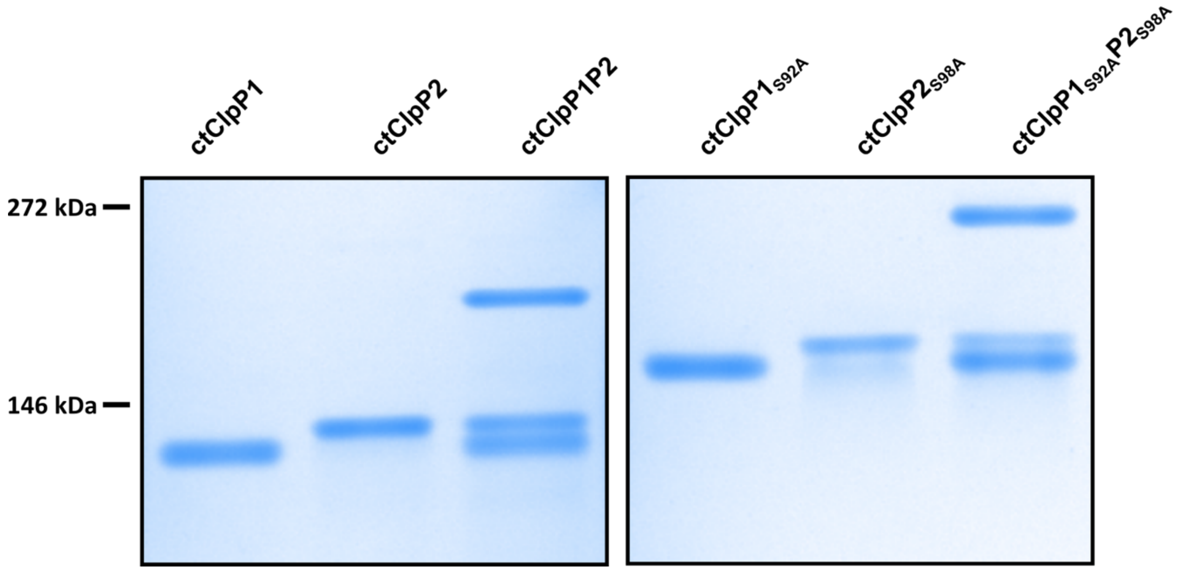

Supplementary Figure S1.

Oligomeric state analyses of native/untagged ctClpP proteins (left) as well as Strep-tagged ctClpP catalytic triad mutant proteins (right).

In native PAGE analyses, stained bands of purified proteins indicated that both ctClpP homologs form homo-heptameric complexes. According to the higher molecular weight of the Strep-tagged proteins, these bands run higher on native PAGE (ctClpP1: 21.16 kDa, ctClpP2: 22.05 kDa, ctClpP1strep: 22.36 kDa, ctClpP2strep: 23.25 kDa). Formation of tetradecameric complexes was only detected by co-incubation of ctClpP1 and ctClpP2. Protein tags or active site mutations did not alter oligomeric states.

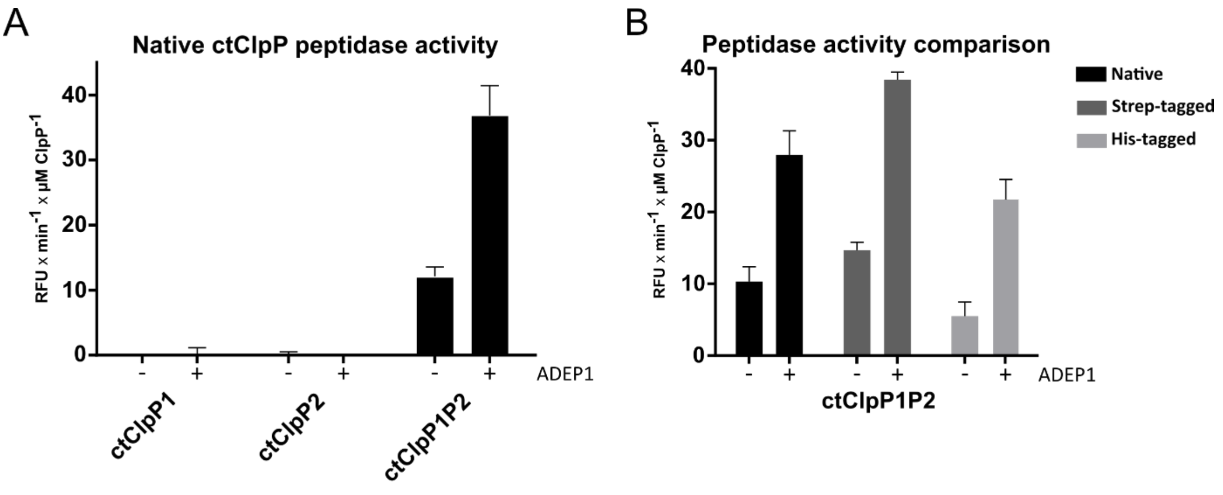

Supplementary Figure S2.

Peptidase activity of native/untagged as well as tagged ctClpP proteins.

A. Suc-LY-AMC degradation of native ctClpP1, ctClpP2 and ctClpP1P2. Both ctClpP homologs did not show any detectable peptidase activity when tested individually. In contrast, native ctClpP1P2 exhibited peptidase activity which could be further enhanced by ADEP1. **B.** Comparison of peptidase activity of tagged ctClpP1P2 complexes compared to untagged, native ctClpP1P2, indicating that C-terminally tagged ctClpP proteins exhibit unimpeded peptidase capabilities relative to native ctClpPs.

Error bars indicate corresponding standard deviations.

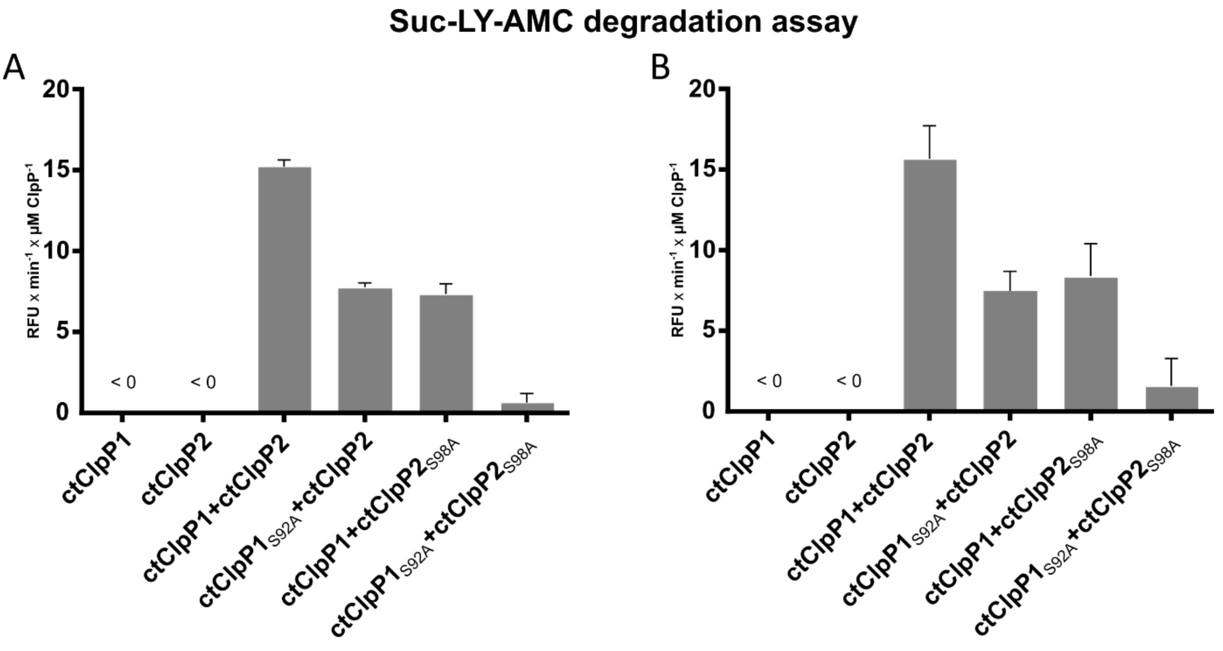

Supplementary Figure S3.

Suc-LY-AMC degradation by ctClpP1, ctClpP2 and corresponding catalytic triad mutants ctClpP1$_{S92A}$/ctClpP2$_{S98A}$.

Independent biological replicates of the assay (**A** and **B**) are shown. Error bars indicate corresponding standard deviations of three independent technical replicates.

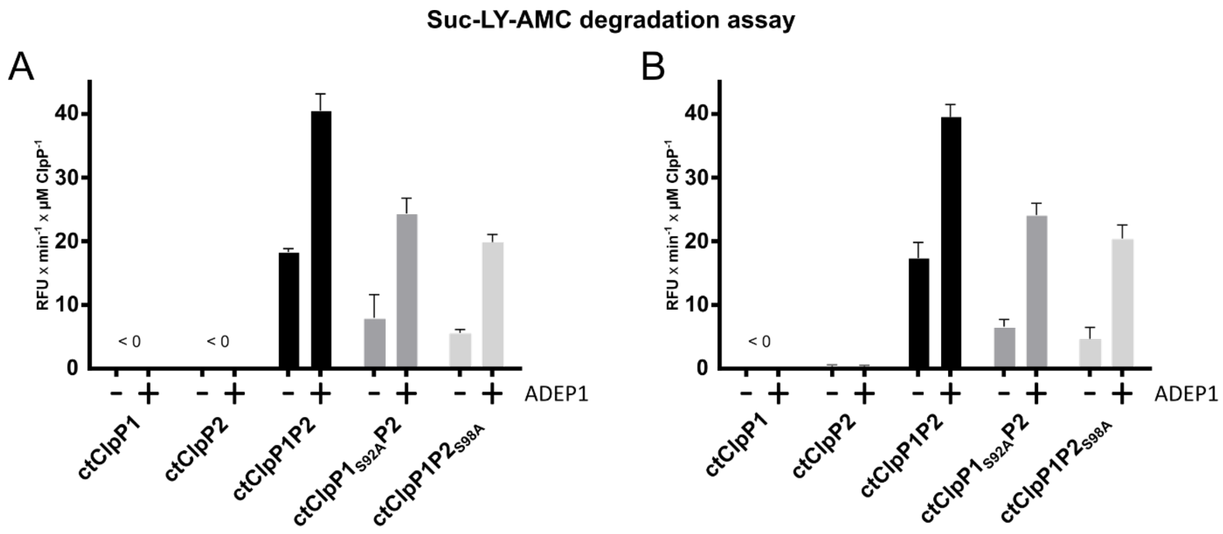

Supplementary Figure S4.

ADEP1-mediated Suc-LY-AMC degradation by ctClpP1, ctClpP2 and corresponding catalytic triad mutants ctClpP1$_{S92A}$/ctClpP2$_{S98A}$.

Independent biological replicates of the assay (**A** and **B**) are shown. Error bars indicate corresponding standard deviations of three independent technical replicates.

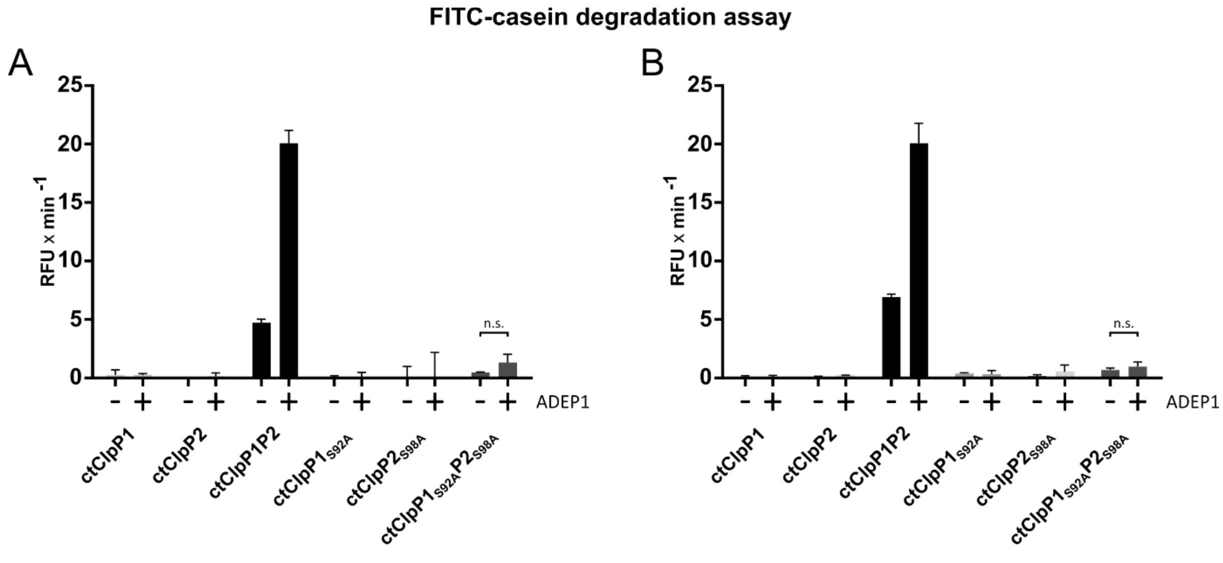

Supplementary Figure S5.

ADEP1-mediated FITC-casein degradation by ctClpP1, ctClpP2 and corresponding catalytic triad mutants ctClpP1$_{S92A}$/ctClpP2$_{S98A}$.

Independent biological replicates of the assay (**A** and **B**) are shown. Error bars indicate corresponding standard deviations of three independent technical replicates.

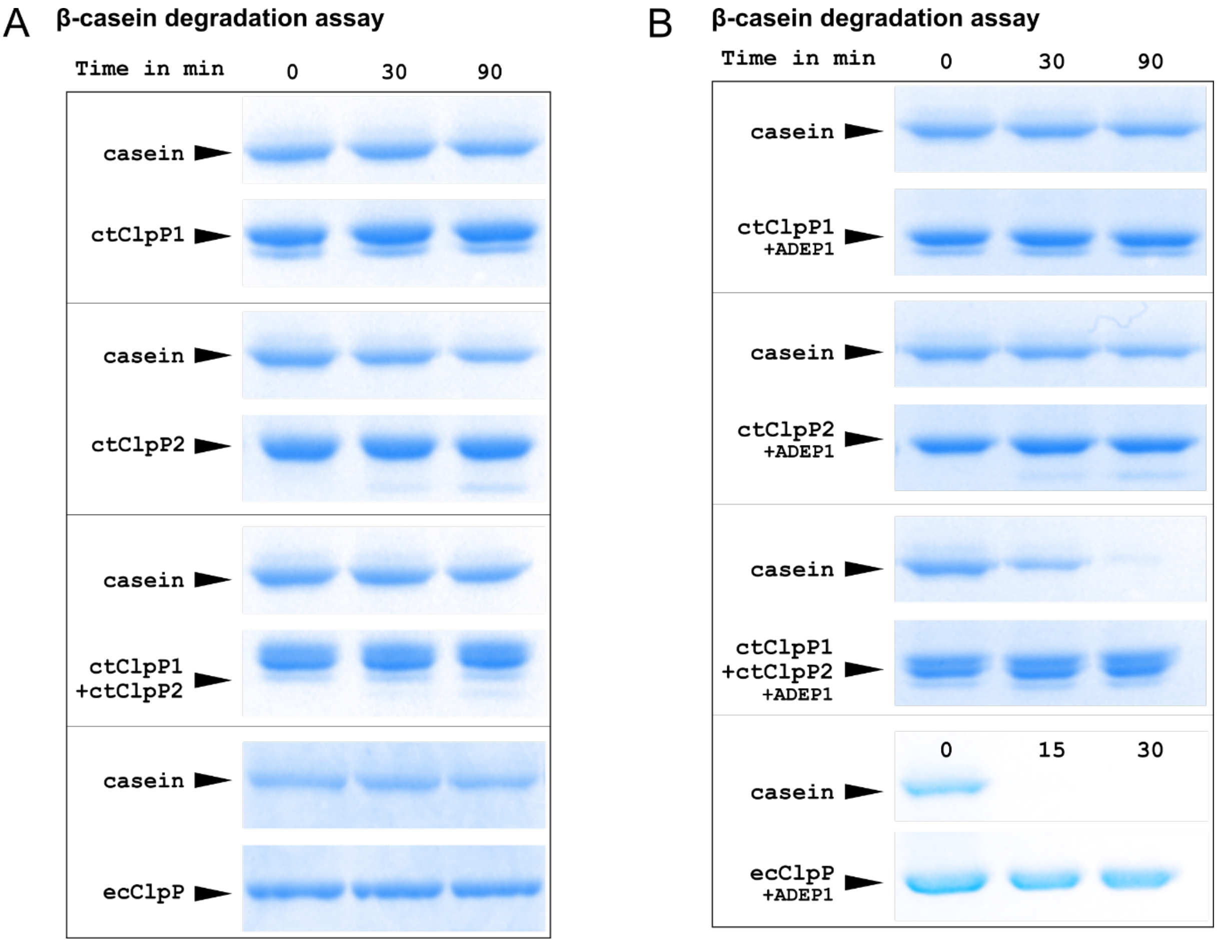

Supplementary Figure S6.

Protease activity of ctClpP proteins using β-casein as a substrate.

A. SDS-PAGE analyses of β-casein degradation by ctClpP proteins. Neither ctClpP1 nor ctClpP2 (alone or in combination), nor ecClpP led to a clear proteolysis of the model protein substrate β-casein in the specified period of time. Data shown are exemplary for at least three independent experiments using at least three independent biological replicates.

B. ADEP1 activated ctClpP1P2 for unregulated proteolysis of β-casein. While ADEP1 did not activate either ctClpP1 or ctClpP2 individually to degrade β-casein, in contrast, ADEP1 clearly activated ctClpP1P2 to degrade β-casein in the given period of time, although slower compared to the highly processive, ADEP1-activated ecClpP. This corroborates our results that either ClpP homolog does not form an active proteolytic complex under these conditions. Data shown are exemplary for at least three independent experiments using at least three independent biological replicates.

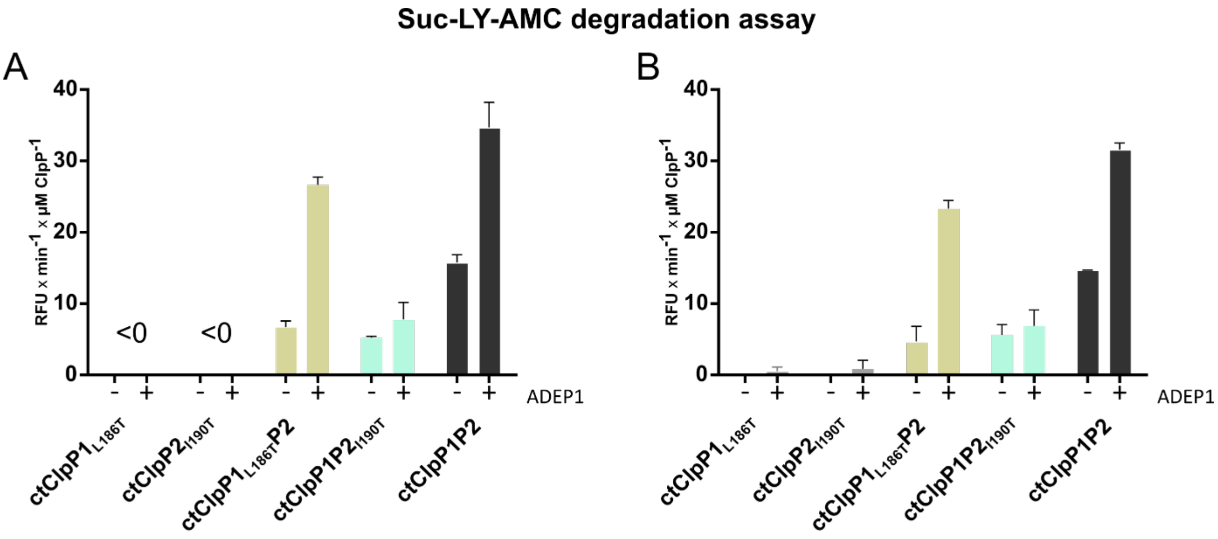

Supplementary Figure S7.

ADEP1-mediated Suc-LY-AMC degradation by ctClpP1, ctClpP2 and corresponding hydrophobic pocket mutants ctClpP1$_{L186T}$/ctClpP2$_{I190T}$.

Independent biological replicates of the assay (**A** and **B**) are shown. Error bars indicate corresponding standard deviations of three independent technical replicates.

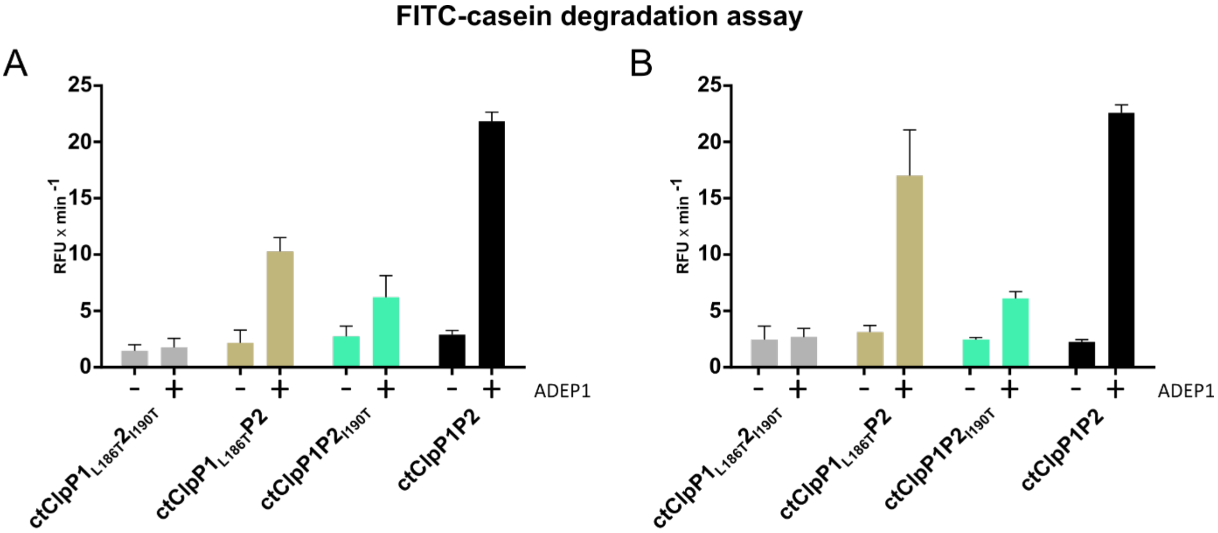

Supplementary Figure S8.

ADEP1-mediated FITC-casein degradation by ctClpP1, ctClpP2 and corresponding hydrophobic pocket mutants ctClpP1$_{L186T}$/ctClpP2$_{I190T}$.

Independent biological replicates of the assay (**A** and **B**) are shown. Error bars indicate corresponding standard deviations of three independent technical replicates.

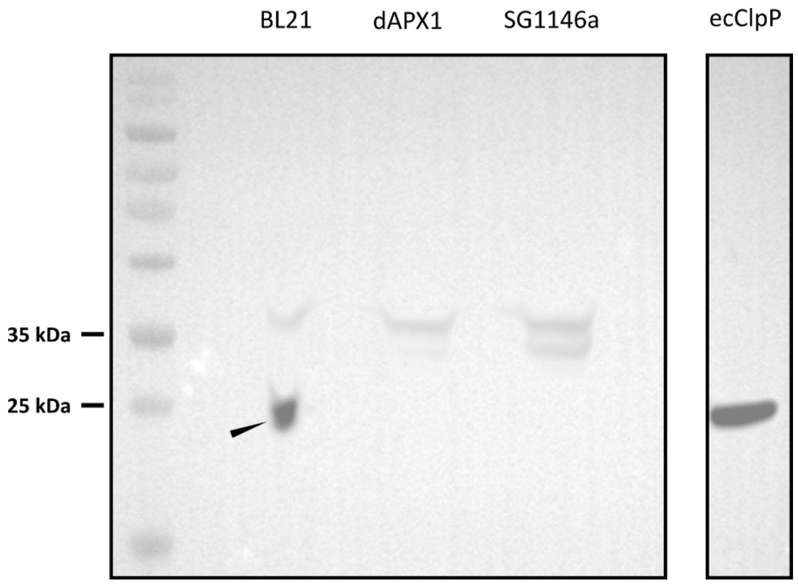

Supplementary Figure S9.

Immunodetection of potential contaminant ecClpP in *E. coli* strains used in this study. Immunodetection of ecClpP in different *E. coli* lysates (strains BL21, dAPX, SG1146a) using anti-ClpP antibodies. Purified ecClpP protein (22.6 kDa) was applied as control (right). Cell lysate of *E. coli* BL21 was tested positive for ecClpP (indicated by arrow). In contrast, endogenous ecClpP was not detected in *E. coli* dAPX1 and SG1146a.

Supplementary Materials and Methods

Native protein purification.

Full length *clpP* genes of *C. trachomatis* D/UW-3/CX (GenBank: NC_000117; *ctclpP1*, orf CT_431; *ctclpP2*, orf CT_706) were amplified via PCR using Phusion DNA polymerase (NEB) using the following primers: *ctclpP1*: forward ATGCATATGCCTGAAGGGGAAATGATG, reverse: ATGGGATCCCTACAAGTCGTTAAAAGAGAAGAGAATC; *ctclpP2*: forward: ATGCATATGACGTTAGTACCATACGTTG, reverse: ATGGGATCCCTAAGACGCAATACT CTTATCTTTTG. *Nde*I and *Bam*HI restriction sites were added to 5' and 3' ends of each PCR product, respectively. PCR products were cloned into pET-11a vector at corresponding *Nde*I and *Bam*HI sites of the polylinker and transformed into *E. coli* dAPX-1. Subsequent protein overexpression and purification were performed in the same manner as for strep-tagged ctClp proteins (see main text) with the following modifications: Filtered lysates of untagged proteins were applied to HiTrap Q XL strong anion exchange (1 ml) columns (GE Healthcare) and eluted using a linear gradient of buffer B (1 M NaCl in buffer A).

β-casein degradation assay.

Degradation of the protein substrate β-casein (Sigma) was carried out in buffer PZ in 150 µl total volume. Each reaction contained 5 µM ctClpP protein (final concentration) and 4 µM β-casein substrate. Where appropriate, a final concentration of 25 µM ADEP1 or equal volume of DMSO was added accordingly. For reference, proteolysis of β-casein by ecClpP (2 µM) in the presence of ADEP1 or the equal volume of DMSO was tested alongside. 10 µl aliquots of each reaction were taken at the indicated time points during incubation at 32 °C. Reactions were halted by heating of the samples under denaturing conditions. Degradation of the substrate was then analysed via SDS-PAGE and subsequent InstantBlue Coomassie protein stain (Sigma). All experiments were performed using at least three independent biological replicates. Data shown are exemplary for at least three independent experiments.
